# Scaled-Down c-Si and c-SiGe Wagon-Wheels for the Visualization of the Anisotropy and Selectivity of Wet-Chemical Etchants

**DOI:** 10.1186/s11671-019-3114-8

**Published:** 2019-08-19

**Authors:** Antoine Pacco, Zheng Tao, Jens Rip, Dennis van Dorp, Harold Philipsen, Frank Holsteyns

**Affiliations:** Imec, Kapeldreef 75, 3001 Leuven, Belgium

**Keywords:** Wagon-wheel mask, c-Si anisotropic etching, Alkaline wet etching, c-SiGe isotropic etching

## Abstract

Wet etching offers an advantage as a soft, damage-less method to remove sacrificial material with close to nanometer precision which has become critical for the fabrication of nanoscale structures. In order to develop such wet etching solutions, screening of etchant properties like selectivity and (an)isotropy has become vital. Since these etchants typically have low etch rates, sensitive test structures are required to evaluate their etching behavior. Therefore, scaled-down single-crystalline Si (c-Si) and SiGe (c-SiGe) wagon-wheels were fabricated. First, the sensitivity of the c-Si wagon-wheels to detect anisotropic behavior of crystalline silicon in the alkaline etchants TMAH and NH_4_OH was demonstrated. Distinctive wagon-wheel patterns, characteristic for each material/etchant pair, were observed by top-down scanning electron microscopy (SEM) after anisotropic wet etching. Similar trends in crystallographic plane-dependent etch rates were obtained for both Si(100) and Si(110) substrates. Secondly, the etching of both c-Si and c-Si_75_Ge_25_ wagon-wheels in a typical selective etchant, peracetic acid (PAA), was evaluated. c-Si_75_Ge_25_ etching in PAA resulted in isotropic etching. Selectivity values were calculated based on two methods: the first by measurement of the sidewall loss of the spokes of the wagon-wheel, the second, indirect method, through measurement of the spoke retraction lengths. Both methods give comparable values, but the latter method can only be used after a certain critical etching time, after which the spoke tips have evolved toward a sharp tip.

**Electronic supplementary material:**

The online version of this article (10.1186/s11671-019-3114-8) contains supplementary material, which is available to authorized users.

## Introduction

Traditionally, the gradual increase of the density of transistors on the integrated circuit semiconductor devices was attained by shrinking the node size. This is technologically and economically no longer sustainable. Therefore, new field effect transistors (FET) architectures like Fin-FET and gate-all-around GAA-FET are introduced [1–3]. The latter offers an advantage over the former because its gate can tune the channel more accurately [4]. Some processes used for the fabrication of these complex 3D features used in most advanced FET transistors and memory cells require extremely selective and isotropic etchants [5, 6].

For the fabrication of GAA architectures comprising one or more vertically stacked horizontal crystalline nanowires, a very selective and isotropic etching of the sacrificial crystalline epitaxial layers is needed. For the release of Si nanowires for example, a Si_x_Ge_1-x_ etchant which leaves the Si nanowires intact, is required.

Therefore, screening and understanding of etchant properties has become vital. Screening of etchants on blanket films gives no reliable information about the (an)isotropy of the material/etchant pair. Visualizing anisotropy is extremely important since the etching of crystalline sacrificial layers can be delayed or even stopped due to the formation of slow etching or so-called ‘blocking’ planes in the lateral trenches. Anisotropy has also been extensively studied for the fabrication of microelectromechanical structures (MEMS) [7, 8] and for the surface texturization of Si in solar applications [9–11].

Principally, two experimental methods have been used, both yielding etch rates as a function of the crystallographic directions of Si. In the first, a silicon sphere or hemisphere with a diameter of some millimeters is etched; anisotropy gives rise to facet formation which, once quantified, yields the etch rates of the different crystal planes [12–14]. In the second and most widespread method, silicon spokes or trenches are patterned on a wafer in a radial manner giving rise to the so-called wagon-wheel shape [15, 16]. The strength of the latter method lies in the fact that many crystallographic faces can be probed in a single wet etching experiment and in its amplification effect. During anisotropic wet etching, the tip of the spokes will retract with a rate proportional to the etch rate of the sidewall of the wagon-wheel spoke, the latter being the etch rate of interest. Due to the geometry of the spoke, the retraction velocity of the spoke tip is significantly higher than the real etch rate of the sidewalls. This relatively large retraction length is thus easier to visualize and quantify than the small sidewall thinning of the spokes. The amplification factor depends on the geometric arrangement of the spokes in a wagon-wheel. Wagon-wheels with more spokes, and thus smaller angles, have larger amplification factors. The wagon-wheels described in literature [15–18] have cm-sized diameters and typically accommodate 180 spokes with an angular width and spacing of 1° resulting in amplification factors of 115. For most applications, high etch rates are desired; therefore, most authors have studied the anisotropic etching of silicon in relatively hot (~ 60–80 °C) and relatively concentrated (~ 12–25 wt.%) alkaline TMAH and KOH solutions. However, there is little known about anisotropic etching in low-concentration alkaline solutions and even less at low temperature. Also, most of the time, only nanometer-removal of semiconductor material is required during the fabrication process of nm-sized structures in most advanced complementary metal oxide semiconductor (CMOS) applications. Therefore, much lower wet etch rates, in the range of a few nanometers/min, are required for most wet etch processes used in very large-scale integration (VLSI). Hence, we propose the miniaturization of the previous generation of wagon-wheels.

In this work, not only scaled-down c-Si but also c-SiGe wagon-wheels were fabricated. As such, the isotropic as well as the selectivity requirements of etchants can be evaluated simultaneously with a high level of accuracy. Since the main asset of the wagon-wheel technique is still the determination of the degree of anisotropy of material/etchants pairs, we will first benchmark our results with those obtained on cm-sized wagon-wheels in previous studies. Then, we will illustrate the utility of this technique for the development of selective and isotropic etchants, specifically for the selective etching of c-Si_75_Ge_25_ with respect to c-Si.

## Experimental/Methods

### Wagon-Wheel Design

The dimensions of the wagon-wheels were chosen with the idea to observe *nanometer*-range sidewall loss resulting in *sub*-*micron* retraction lengths. The wagon-wheel dimensions are based upon a balance between the following three boundaries:

1. The photolithographic technique setting a constraint on the minimal critical dimension (CD), which is the wagon-wheel (inner) spoke width.
2. The imaging technique defining a maximum practical field of view (FoV), and thus a maximum wagon-wheel diameter.
3. The maximum number of spokes that can be arranged in the circular wagon-wheel pattern, or accordingly, the minimum spoke wedge angle, defining the maximum amplification factor.

**Fig. 1:**
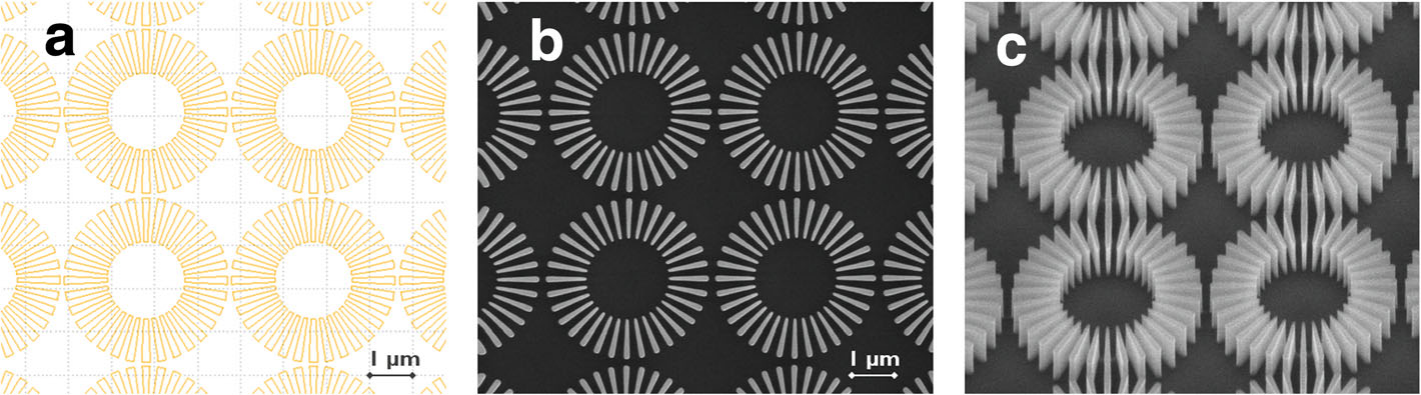
Wagon-wheels as designed on mask (**a**) and after the nanofabrication process: top view SEM (**b**), tilted view SEM (**c**)

Considering this, the inner spoke width was set at 90 nm (CD), the wagon-wheel diameter at 3.8 μm (FoV), and 32 spokes were arranged in a circular pattern. This wagon-wheel design results in spoke angles of 5.6° and an amplification factor of about 20. A dedicated mask was designed for this purpose (Fig. 1a). The dimensions are compared with those from previously fabricated wagon-wheels by Wind et al. (see Table 1). The wagon-wheels are aligned in vertical and horizontal directions with a pitch of 3.9 μm, leaving 100 nm space in between two wagon-wheels (Fig. 1b). The fabrication of these wagon-wheels will be described in the next section.

**Table 1:** Scaled-down wagon-wheel dimensions. Previously reported wagon-wheel dimensions are shown in the last column

	This work	Wind et al. (ref. [15])
Wagon-wheel diameter	3.8 μm	16 mm
Spoke length	1 μm	8 mm
Spoke height	600 nm	130 μm
Spoke width (outer)	180 nm	140 μm
Spoke width (inner)	90 nm	2 μm
Spoke angle/space	5.6°/5.6°	1°/1°
Number of spokes	32	180
Amplification factor	20×	115×

### Wagon-Wheel Fabrication

Crystalline silicon (c-Si) wagon-wheels were fabricated on standard p-type (B-doped, 1–100 Ohm cm) 300 mm Si(100) and Si(110) wafers. The patterning process consisted of the deposition of a hardmask stack composed of (from top to bottom) 30 nm silicon nitride, 160 nm amorphous carbon (APF), 20 nm silicon nitride, and 30 nm amorphous Si. A 193-nm immersion lithography was used to transfer the pattern into the photoresist. After the photoresist was developed, the wagon-wheel pattern was etched using a plasma etch which included a photoresist and APF strip. The bottom layer of the hardmask stack, being the SiN layer, was removed using hot phosphoric acid (6 min. 85 wt.% H_3_PO_4_ at 160 °C) or hydrofluoric acid (5 min 10 wt.% HF). Figure 1c shows a tilted SEM view of the fabricated wagon-wheels.

Crystalline silicon-germanium wagon-wheels (c-Si_75_Ge_25_) were also fabricated on standard p-type (B-doped, 1–100 Ohm cm) 300 mm Si(100) or Si(110) wafers. Before patterning, a layer of approximately 600 nm undoped Si_75_Ge_25_ was deposited epitaxially. After this, the same patterning steps as for the Si wagon-wheels were followed resulting in c-Si_75_Ge_25_ wagon-wheel spokes.

### (An)Isotropic Wet Etching Experiments

Prior to the (an)isotropic wet etching tests, a SPM clean (5 min H_2_SO_4_:H_2_O_2_ 3:1 at 60 °C), aimed for the removal of organic residues was performed and, following this, the oxide layer was removed during a 2-min immersion in aqueous 1 wt.% HF solution. Immediately after the SPM and HF cleaning processes, etchant anisotropy is assayed by placing the test pattern in an unstirred sample of the etchant at room temperature (RT). TMAH and NH_4_OH etchant solutions were prepared by dilution of 25 wt.% TMAH or 29 wt.% NH_4_OH. The peracetic acid (PAA) solutions were prepared by mixing 9.5 parts of H_2_O_2_ (30 wt.%), 11 parts of acetic acid (98 wt.%), and 0.1 parts HF (49 wt.%). This etching solution is known to selectively etch Si_x_Ge_1-x_ alloys over pure Si [19, 20]. PAA, which acts as the oxidizing species for SiGe etching, is formed by reaction of the acetic acid with the peroxide with HF as catalyst. However, a certain time is needed to reach equilibrium; therefore, the solutions were aged for 1 week. Etchant anisotropy and selectivity was assayed by placing the test patterns in an unstirred sample of the etchant at RT. Immediately after etching, the samples were rinsed for 30 s in deionized water and subsequently dried with nitrogen gas.

## Results and Discussion

### Anisotropic Etching of c-Si(100) and c-Si(110) Wagon-Wheels in TMAH

**Fig. 2:**
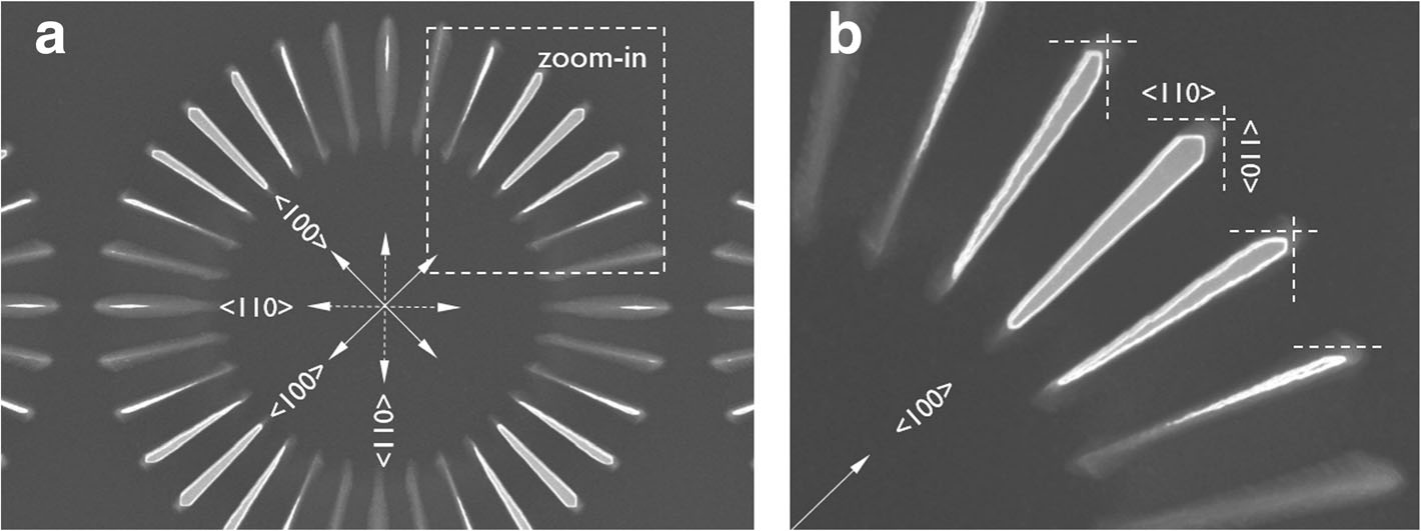
**a** TD SEM of a Si(100) wagon-wheel etched in low-concentration TMAH (5 wt.%) at RT and zoom-in (**b**) showing the development of the faster etching {110} planes/facets, as indicated by the dashed lines

When silicon wagon-wheels fabricated on a Si(100) wafer are etched in low-concentration TMAH (5 wt.%) at RT, the following observations can be made (Fig. 2): first, the characteristic fourfold symmetry of a Si(100) wafer is revealed through the anisotropic etching of the wagon-wheel. Second, the orientation-dependent etch rate of different crystallographic planes can visually be deduced: the relatively fast etching spokes of the wagon-wheels are those defined by {110} and vicinal {110} sidewall planes, while the slower etching spokes are defined by the {100} and vicinal {100} sidewall planes. Besides this main observation that the etch rate order of Si in low concentration and RT TMAH follows *R*_(110)_ > *R*_(100)_, other anisotropic effects could be discerned: for instance, the four spokes corresponding to the four {110} planes are not the fastest etching spokes, those are, more precisely, each time the two vicinal spokes of these {110} planes. Accordingly, the etch rate around {110} is split into two equivalent maxima, and the {110} planes are local minima. This corresponds to similar observations made by [21–23] wherein the lower etch rate of the {110} planes is attributed to a blocking effect by the TMA^+^ ions.

Another result of the anisotropic etching is the particular shape of the outer spoke ends of the four {100} spokes. It is known that the *fastest* etching planes will be revealed for convex surfaces. Initially, the spoke ends are convex surfaces, consequently after a certain etching time, the faster etching {110} planes are revealed, forming facets at the outer spoke ends. This is most obvious for the spokes along the <100> directions (zoom-in b of Fig. 2).

**Fig. 3:**
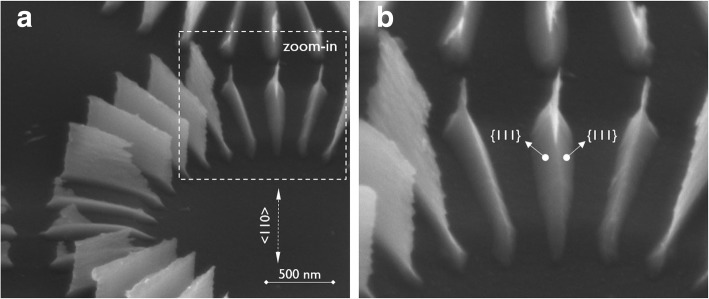
**a** Tilted SEM image of a Si(100) wagon-wheel etched in low-concentration TMAH (5 wt.%) at RT and zoom-in (**b**) showing the slanted {111} planes

For a *concave* surface, however, the *slowest* etching planes will be revealed. During etching of the wagon-wheel spokes, the Si(100) substrate surrounding the spokes is also etched. This base-substrate transition is a concave surface; therefore, the slowest etching planes, being the {111} planes, should be revealed. These {111} planes appear aligned with the <110> directions for Si(100) substrates. In fact, it can be seen in Fig. 3b that the {111} planes were revealed after etching in TMAH, forming a base with slanted {111} planes for all spokes along the <110> directions.

**Fig. 4:**
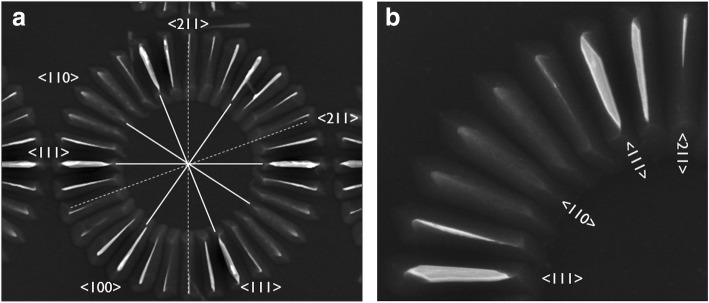
**a** TD-SEM of a Si(110) wagon-wheel etched in low-concentration TMAH (5 wt.%) at RT and zoom-in (**b**) showing the facet formation on the <111> oriented spokes. The main crystallographic directions (or equivalent planes) are represented by solid lines, the higher index planes by dashed lines

Similar to the Si(100) wagon-wheels, Si(110) wagon-wheels were etched in low-concentration TMAH (5 wt.%) at RT. Instead of the fourfold symmetry of a Si(100) wafer, the twofold symmetry of the Si(110) is revealed. The crystallographic orientations of some of the {100}, {110}, {111}, and {211} planes are assigned in Fig. 4a. One of the benefits of using Si(110) substrates for the evaluation of anisotropy is the presence of vertical {111} planes, which are represented by the sidewalls of the {111} spokes of the wagon-wheels. As can be seen in Fig. 4, these are the slowest etching planes. The fastest etching planes seem to be the {110} and the {211} planes. Intermediate etch rates are found for the {100} planes. Hence, *R*_(110)_ ~ *R*_(211)_ > *R*_(100)_ > *R*_(111)_, in line with results obtained on Si(100).

It can be seen in Fig. 4b that the ends of the slowest etching {111} spokes evolve from a rounded shape toward an arrow-like shape, forming an asymmetric parallelogram. The underlying cause for the formation of these facets is the faster etching of the {110} and the {211} planes.

TMAH and TMAH/IPA mixtures are well-studied and commonly used etchants for the fabrication of MEMS structures for which high etch rates and tuning of anisotropy are required. Accordingly, most research on Si etching in TMAH has been done at higher temperatures and concentrations. Typical concentrations range from 10 to 25 wt.% TMAH and at temperatures ranging from 60 to 90 °C [12–14, 23]. A few research groups performed etching tests at low concentrations around 5 wt.%, like in our work, but they still used high temperatures 60–90 °C [24–26]. The *R*_(110)_/*R*_(100)_ ratio lies typically around 2 for high concentrations and high temperatures and seems to increase with *decreasing* TMAH temperature (Additional file 1: S1). Since this study focusses on nanoscale etching applications, high etch rates are not pursued. Therefore, low (RT) temperatures were chosen in order to have an acceptable time window for the observation of etching phenomena and to avoid the complete dissolution of structures. The same order of velocities, *R*_(110)_ > *R*_(100)_, is observed in this study, done at RT and 5 wt.% TMAH, but the calculated values of the *R*_(110)_/*R*_(100)_ ratio are well above 2 (see also reference [27]). Thus, this confirms the trend that this anisotropic ratio increases with *decreasing* TMAH temperature. A detailed mechanistic explanation of this observation, including kinetic and atomistic aspects, lies beyond the scope of this work. However, based on the above comparisons for the etching of silicon in TMAH, it can be concluded that the scaled-down wagon-wheels provide the required sensitivity to detect and compare the anisotropic behavior of etchants.

### Anisotropic Etching of c-Si(100) and c-Si(110) Wagon-Wheels in NH_4_OH

**Fig. 5:**
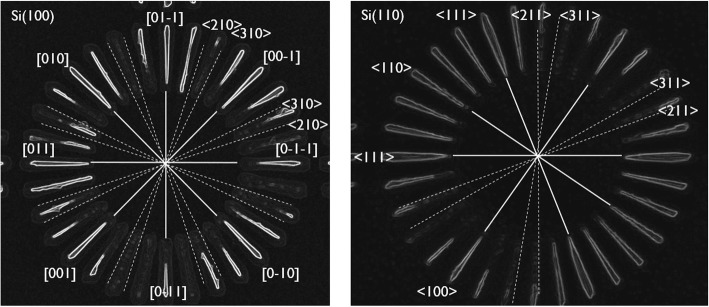
TD SEM image of a Si(100) (left) and a Si(110) wagon-wheel (right) etched in low-concentration NH4OH (0.4 wt.%) at RT. Specific crystallographic directions are represented by [ ], a family of equivalent directions by < >. The main crystallographic directions (or equivalent planes) are represented by solid lines, the higher index planes by dashed lines

Si(100) wagon-wheels as well as Si(110) wagon-wheels were etched in low-concentration (0.4 wt.%) ammonium hydroxide (NH_4_OH) at RT. In the former (Fig. 5, left), the fourfold symmetry of a Si(100) wafer is revealed. It is clear that the spokes along the <210> and <310> directions, which are theoretically at 18.4° and 26.6° with respect to the <110> directions, best represented by the third spoke (counting from the top ‘northern’ spoke) with sidewalls at 19.7° and 25.3°, are the fastest etching spokes. The {110} spokes are slower etching compared to {100} and facets develop at the outer ends of these spokes. These facets are probably the fast etching {210} and {310} planes and may contribute to an overall faster apparent etch rate of the {110} spokes, especially for longer etching times. Thus the observed etch rate follows *R*_(310)_ ~ *R*_(210)_ > *R*_(100)_ ~ *R*_(110)_.

For the Si(110) wagon-wheels (Fig. 5, right), the twofold symmetry around the (100) and (110) plane is revealed through anisotropic etching in NH_4_OH. The spokes along the <111> directions, with {111} sidewalls, appear as the slowest etching spokes or planes. The fastest etching spokes are defined by the high index {211} and {311} planes. The {110} and {100} have intermediate etching rates. Hence, the Si(110) results are in line with the Si(100) results in NH_4_OH. Also for the Si(110) wagon-wheels, faceting of the spokes is noticeable, especially at the outer ends of the {111} and {100} spokes. The facets are probably the development of the faster etching {211} and {311} planes.

In literature, limited information is available for the etch rate anisotropy of NH_4_OH. However, aqueous solutions of NH_4_OH have also been used as anisotropic etchants [28], with similar properties as other OH^-^ containing Si etchants. The benefit is that it does not contain metals (like K^+^, Na^+^, Cs^+^, ...). Therefore, NH_4_OH is an IC-compatible etchant worth investigating. Schnakenberg et al. showed that the *R*_(111)_/*R*_(100)_ etch rate ratio for a wagon-wheel type etch pattern etched in 3.7 wt.% NH_4_OH at 75 °C is approximately 0.04 and the *R*_(110)_/*R*_(100)_ etch rate ratio 0.3 [28]. The later result compares well with our estimated etch rate ratio of 0.5 for *R*_(110)_/*R*_(100)_.

From our results, it is clear that the etching of Si in NH_4_OH gives dissimilar wagon-wheel etch patterns compared to TMAH. Although there is a small difference in the [OH^−^] for both etching solutions (~ 0.12 M vs. ~ 0.55 M), both the etching in TMAH and in NH_4_OH were performed at the same temperature (RT). The only remaining difference is the counter-cation: the bulkier (CH_3_)_4_N^+^ compared to the smaller NH_4_^+^ cation. It has been pointed out that cations in the etchant solution could adhere to the surface, thus selectively blocking different hydroxyl-terminated Si surface sites associated with the different etching planes [29, 30]. Whenever the etching rates of different planes are affected differently, the anisotropy will change.

### Selective Etching of Si_75_Ge_25_ Toward Si

**Fig. 6:**
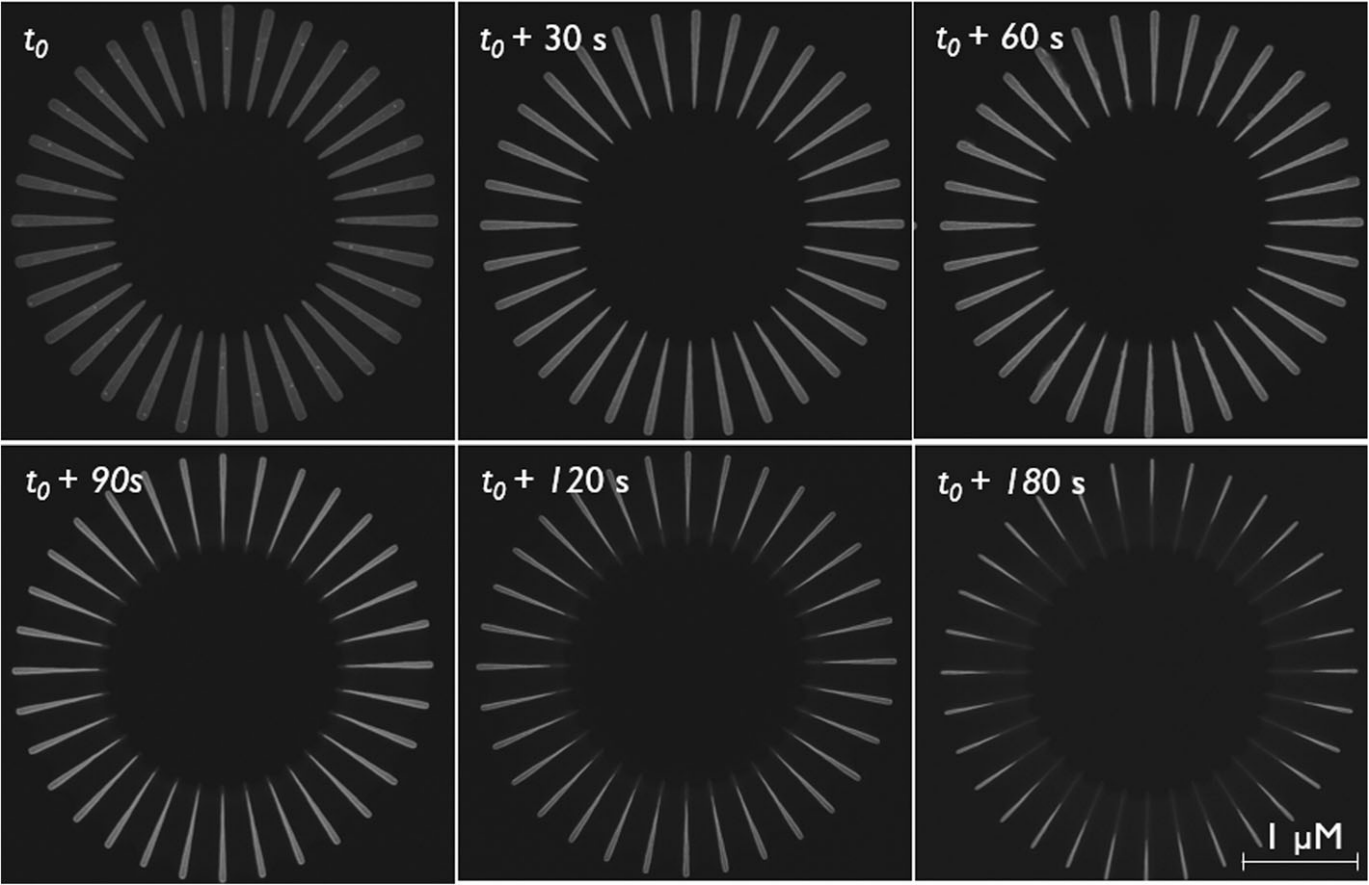
Etching time series of Si75Ge25(100) wagon-wheels in PAA-solution

**Fig. 7:**
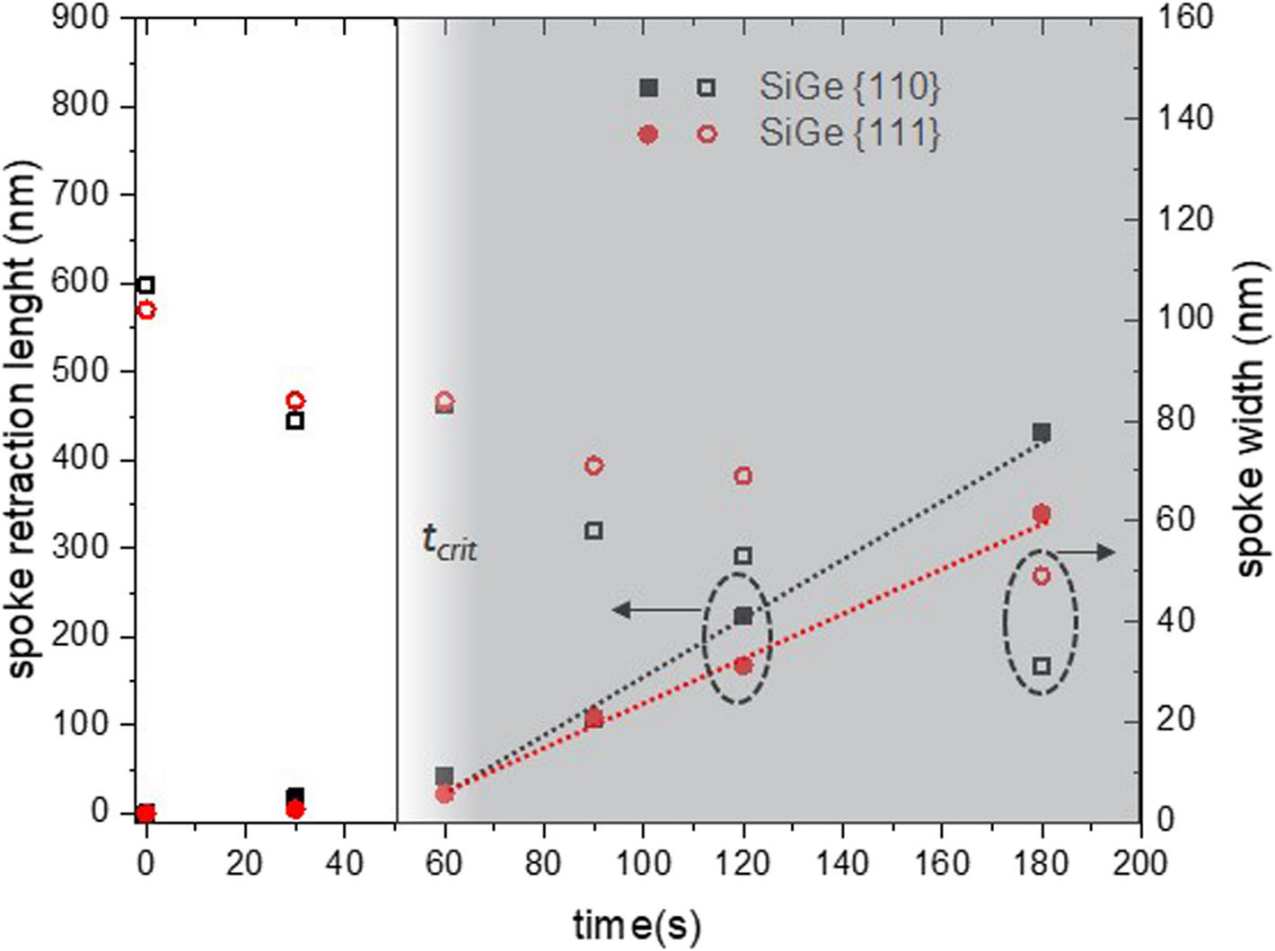
Etching of Si75Ge25 spokes: the spoke width decreases gradually, but only after a certain tcrit., the spoke tips start to retract and the measured retraction lengths Si75Ge25 can be fitted linearly a.f.o. etching time (gray-shaded area)

In this section, we will demonstrate and discuss the potential of the scaled-down wagon-wheels for the evaluation of the etch rate and selectivity of etchants. The Si/Si_75_Ge_25_ pair was selected as a model system since it is representative for the formation of GAA structures whereby the sacrificial c-Si_75_Ge_25_ interlayers should be etched isotropically and selectively toward the c-Si nanowires. Etching is performed in a selective etchant prepared by a mixture of HF, H_2_O_2_, and CH_3_COOH. It is known that this mixture will form peracetic acid (CH_3_CO_3_H) due to the acid-catalyzed reaction between the peroxide and the acetic acid [31, 32]. After a certain aging time, the equilibrium concentrations are reached. The so formed PAA is an effective and selective oxidizer of Si_75_Ge_25_. After the selective oxidation of SiGe, the SiGe oxides will be dissolved by HF in a second, diffusion-limited reaction.

c-Si_75_Ge_25_ wagon-wheel samples were dipped in the PAA solution for increasing times (*t*_0_+ 30 s, + 60 s, ...+ 180 s) and the etching of the wagon-wheel spokes was monitored by subsequent top-down SEM measurements. The widths of the spokes can be reliably measured by our conventional SEM if they are not smaller than 10 nm. The results are shown in the time series in Fig. 6. Initially, the wagon-wheel spokes are thinned due to the etching of their sidewalls. All Si_75_Ge_25_ spokes are thinned equally, proving the etching to be isotropic. After approximately 90 s, the spoke tips start to retract, suggesting the initiation of the amplification effect. We observe that this amplification effect starts to manifest itself only after the spoke tips have evolved toward a sharp tip. At *t*_0_, the tips of the spokes are still rounded. Due to the gradually converging sidewalls during the initial etching stage of the spokes (*t* < 90 s), the rounded tip transforms to a merely sharp tip and the spokes start to retract (see also Additional file 1: S2). This finding is clearly illustrated for the wagon-wheel spoke shape at *t* = 180 s: roughly half of the spoke has been etched due to tip retraction (∆l is roughly 450 nm). However, there is still some SiGe left, at least at the broadest end of the spokes, since the sidewalls only retracted by an amount ∆w~∆l/20 = 22.5 nm at both sides. Consequently, after a critical time (*t*_*crit*_), the retraction length (∆l) can be used to indirectly calculate the etch rate of the Si_75_Ge_25_ spokes. However, before this *t*_*crit*_, the etch rate can only be calculated by direct measurement of sidewall loss (∆w) which is difficult to measure. A comparison of the etch rates of Si and SiGe in PAA obtained by direct measurement of the sidewall loss and indirect measurement of the spoke retraction is shown in Table 2. The etch rates were obtained by the slope of the decreasing sidewall widths versus time and the slope of the increasing spoke retractions lengths versus time. The latter slope was calculated using the data points after *t*_*crit*_ as shown in Fig. 7. The sidewall widths seem to decrease linearly, at least to the limit of observation of our conventional SEM, which is down to approximately 10 nm. Down to these feature sizes, we did not observe any striking changes in the etch rate during the gradual thinning of the spokes (Figs. 7 and 9).

**Table 2:** Overview of the etching rates of Si and Si75Ge25 in PAA obtained by direct measurement of the sidewall-loss and indirect measurement of the spoke retraction. The errors represent the standard error of the slope. Last column shows the calculated selectivity ratios of Si75Ge25/Si

	Si etch rate (nm/min)		SiGe etch rate (nm/min)		Selectivity (SiGe/Si)	
	Sidewall	Retraction	Sidewall	Retraction	Sidewall	Retraction
{111}	0.66 ± 0.04	0.71 ± 0.05	8.3 ± 0.9	7.8 ± 0.4	12.6	11.0
{110}	0.73 ± 0.03	0.76 ± 0.09	12.3 ± 0.7	10.8 ± 0.3	16.8	14.2

Besides obtaining etch rates for the SiGe etchant, we verified the isotropic behavior of the etchant. It is clear that all differently oriented spokes etch at the same etch rate, i.e., isotropically. This thus points toward a process whose reaction rate is controlled by the oxide dissolution rate and not by the Si_75_Ge_25_ oxidation rate. Oxide dissolution is diffusion limited, with low activation energies, and is not prone to anisotropic behavior.

Results obtained on c-Si_75_Ge_25_(100) wagon-wheels were validated with c-Si_75_Ge_25_(110) wagon-wheels. Like explained for the silicon wagon-wheels, one benefit of using (110) substrates is the additional presence of vertical {111} planes, represented by the sidewalls of the {111} spokes of the wagon-wheels. These are typically the slowest etching planes/spokes; thus, a careful observation of those spokes is necessary for a complete image of the anisotropy of the Si_75_Ge_25_-PAA etchant pair. The results (see Additional file 1: S3.1) are in line with the etching results obtained with the Si_75_Ge_25_ (100) wagon-wheels (Fig. 6). The etching proceeded gradually, first by thinning followed by spoke tip retraction. A slight non-uniformity in the retraction lengths of the differently oriented spokes can be observed at the longest etching time (*t* = 180 s). However, since there is no clear trend, i.e., a specific angle dependence of ∆l, this was not attributed to anisotropy. We attribute this merely to a larger variation (inter- and intra-spoke) of the spoke widths after fabrication. Indeed, it can already be seen in the reference picture (*t*_0_) that the sidewalls are not perfectly straight. This sidewall roughness is probably due to relaxation defects of the epitaxially deposited Si_75_Ge_25_ layer on a (110) substrate. In summary, both the c-Si_75_Ge_25_ (100) and (110) wagon-wheels are etched isotropically in the PAA solution, being a benefit for the fast and complete removal of c-Si_75_Ge_25_, used as a sacrificial material, since it will not tend to form any blocking planes.

Wet etching in PAA was repeated for c-Si wagon-wheels. The purpose of these tests is to verify the selectivity of the etchant solution toward silicon. The samples were dipped in an identical PAA solution for increasing times (*t*_0_+ 15 min, + 30 min, ...+ 90 min). Note that the etching times are in *minutes* and not in seconds as for the Si_75_Ge_25_ wagon-wheels. These extended etching times are intended to observe any Si etching even if the purpose of this etchant is to preserve the silicon.

**Fig. 8:**
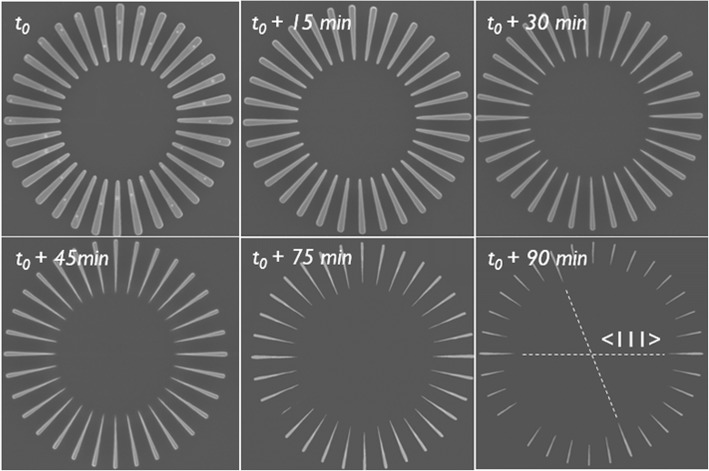
Etching time series of Si(110) wagon-wheels in PAA-solution. Note the slightly slower etch rate of the <111> spokes compared to the other directions, most discernible in the image *t*_0_+ 90 min, and indicated by the dashed lines

**Fig. 9:**
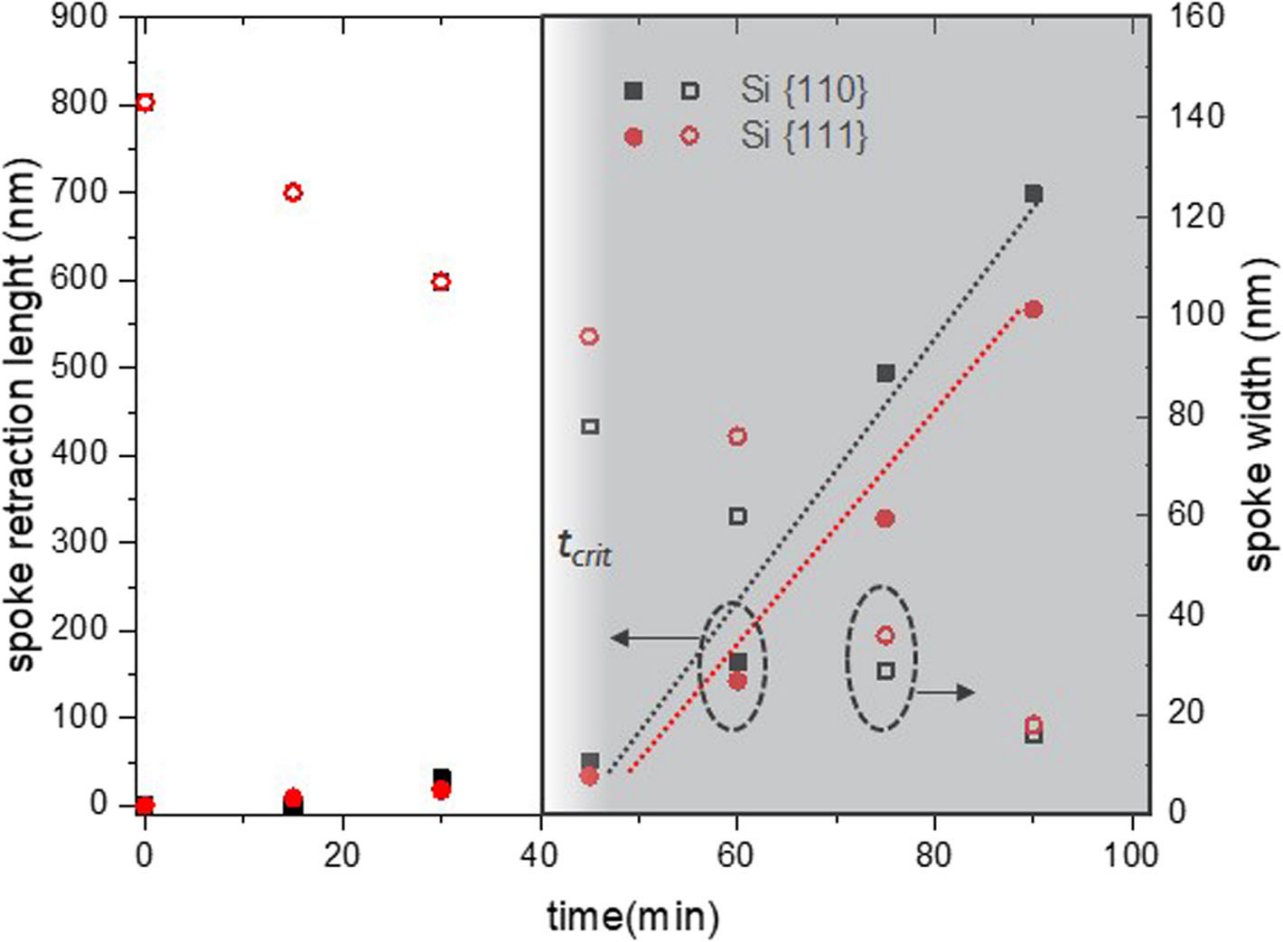
Etching of Si spokes: the spoke width decreases gradually, but only after a certain tcrit., the spoke tips start to retract, and the measured retraction lengths can be fitted linearly a.f.o. etching time (gray-shaded area)

Although the etching times were different, a similar observation as for the Si_75_Ge_25_ wagon-wheels spokes was made: initially, the silicon spokes are gradually thinning down due to their relatively slow sidewall etching, then after a time, *t*_*crit*_, in this case after approximately 45 min, the spokes start to retract relatively fast due to the amplification effect (Figs. 8 and 9). In all cases, the etching seems isotropic. The time series obtained with c-Si(100) wagon-wheels (see Additional file 1: S3.2) are in line with the time series obtained with the c-Si(110) wagon-wheels (Fig. 8).

Calculated etch rates are shown in Table 2. Both measurement methods give comparable etching rates with only a 7% and 4% difference in the etch rate values of the {111} and {110} planes, respectively. The values of the Si etching rate are all < 1 nm/min. Holländer et al. measured Si(100) etch rates of ~ 10 nm/min with HF:H_2_O_2_:CH_3_COOH 1:2:3 with a HF concentration of 1.6 wt.% and Wieser et al. measured etch rates of ~ 3 and 5 nm/min for undoped Si(111) and Si(100), respectively with BHF:H_2_O_2_:CH_3_COOH 1:2:3 solutions with a HF concentration of 1 wt.% [33, 34]. Our values compare well with those reported values, considering that the HF concentration in our tests are lower ([HF] = 0.25 wt.%). Our results also suggest a slightly lower etching rate of the {111} planes, measured on Si(110) substrates, compared to the {110} planes, measured on Si(100) substrates. These quantitative results point toward a very low etching anisotropy of Si in PAA which is hardly observable by the top-down SEM images. For the sake of clarity, the {111} planes of the wagon-wheel at *t*_0_+ 90 min in Fig. 8 are indicated and it can be noticed that the {111} spokes are slightly broader and longer than the surrounding spokes. This demonstrates again that these scaled-down wagon-wheels are sensitive to detect very faint differences in the crystallographic plane-dependent etch rates (‘anisotropy’) of etchants.

The selectivity ratios of the Si_75_Ge_25_/Si pair in PAA were extracted from the etching rates in Table 2. The selectivity ratios of Si_75_Ge_25_(111)/Si(111) range between 11.0 and 12.6 while the Si_75_Ge_25_(110)/Si(110) ratios are slightly higher, between 14.2 and 16.8. These values are slightly lower than the reported values from Holländer et al. who claim selectivities around ~ 20 [33]. This can be attributed to the higher SiGe etch rates (11–17 nm/min) due to the dynamic process conditions (wafer rotation) in contrast to our static process conditions (no stirring) in which case the SiGe etch rates were ranging between 7.8 and 12.3 nm/min. Interestingly, this confirms the observed isotropic etching of SiGe in PAA: since the reaction rate is kinetically controlled (by stirring or rotation), the rate determining step (RDS) is most probably the diffusion controlled SiGe-oxide dissolution by HF.

## Conclusions

Scaled-down wagon-wheels with a diameter of 4 μm and 32 spokes exposing the different crystallographic planes were fabricated on 300-mm-diameter wafers. The structures were patterned on Si(100), Si(110), Si_75_Ge_25_(100), and Si_75_Ge_25_(110) substrates allowing the observation of the etching of the three main crystallographic orientations of c-Si and c-Si_75_Ge_25_ ({111}, {110}, and {100}) as well as higher index planes. The structures proved to be valuable for the evaluation of the isotropic or anisotropic behavior of etchants by simple inspection by TD SEM. Various alkaline as well as acidic etchants were evaluated by image analysis of their characteristic wagon-wheel etching pattern. Trends in etching ratios were in good agreement with previous works. In TMAH, the plane-dependent etching rate of silicon follows the order: *R*_(110)_ ~ *R*_(211)_ > *R*_(100)_ > *R*_(111)_. In NH_4_OH, on the other hand, the etching rate follows the order: *R*_(310)_ ~ *R*_(210)_ > *R*_(100)_ ~ *R*_(110)_ > *R*_(111)_. Besides the relative etching rates of the main crystallographic planes, other anisotropic features, like facets, were observed, indicating that the structures are very sensitive to changes in the anisotropic properties of the etchant.

In addition to their capacity for the revelation of the (an)isotropy of etchants, these wagon-wheel structures also demonstrate their benefit for the assessment of the selectivity of etchants. For this purpose, the system PAA/Si/Si_75_Ge_25_ was assessed in terms of Si_75_Ge_25_ etching, selective toward Si. Selectivity values were obtained by two methods: the first by measurement of the sidewall loss of the spokes; the second, indirect method, through measurement of the spoke retraction lengths. It was shown that the latter method could only be used after a certain critical etching time, after which the spoke tips have evolved toward a seemingly sharp tip.

In conclusion, scaled-down wagon-wheels can be used as lab-scale vehicles for the swift evaluation of anisotropy and selectivity of material/etchant pairs. The structures also have the potential to be used as high-throughput short loop test structures for the screening of etchants on 300 mm wafer wet processing tools. In addition, due to their small size, these wagon-wheels could be used for future in-situ etching studies, using liquid cell environmental electron transmission microscopy ETEM.

## Additional file

Additional file 1: **S1.** Anisotropic etch rate ratio (R_(110)_/R_(100)_) as a function of temperature. Anisotropic etch rate ratios, R_(110)_/R_(100)_, for various temperatures and TMAH concentrations (in wt.%) extracted from other studies [Tabata, 1992; Sato, 1999; Shikida, 2000 & 2001; Pal, 2009]. In all reported cases of etching of c-Si in TMAH, there seems to be a negative correlation between the anisotropic etch rate ratio, R_(110)_/R_(100)_ and the temperature. **S2.** Spoke retraction and onset of the amplification effect. This schematic illustrates the gradual wet etching of a wagon-wheel spoke as a function of the etching time and the onset of the spoke retraction. The amplification effect initiates only after the spoke tip is sharpened (b), after which the retraction length (∆l) can be used as an approximation for the sidewall loss (∆w), and thus for the etch rate of the sidewall plane. Before situation (b), the etch rate can only be calculated by direct measurement of the sidewall loss (∆d). (a) Top view of the spoke before wet etching. (b) Spoke after wet etching resulting in sidewall loss (∆d) and in tip sharpening. The dotted line in ‘b’ represents the starting spoke contour of spoke ‘a’. Note that the spoke length did not significantly decrease. (c) Further wet etching results in a significant spoke length retraction (∆l) that can be correlated to the sidewall loss (∆w) by the formula ∆w = ∆l . sin(α/2). The dotted line in ‘c’ represents the contour of spoke ‘b’. **S3.1.** Etching time series of c-Si_75_Ge_25_(110) wagon-wheels in PAA-solution. **S3.2.** Etching time series of c-Si(100) wagon-wheels in PAA-solution. (DOCX 495 kb)

## Data Availability

All data are fully available without restriction.

## Abbreviations

AA: acetic acid
BHF: buffered hydrofluoric acid
CD: critical Dimension
CMOS: complementary metal oxide semiconductor
c-Si: single-crystalline silicon
c-SiGe: single-crystalline silicon-germanium
ETEM: environmental electron transmission microscopy
FinFET: fin field effect transistor
FoV: field of view
GAA: gate all around
HF: hydrofluoric acid
MEMS: microelectromechanical systems
PAA: peracetic acid
*R*_(xyz)_: etch rate of a (xyz) plane
RDS: rate determining step
SEM: scanning electron microscopy
TMAH: tetramethyl ammonium hydroxide
VLSI: very large-scale integration
