# Supplementary material for: Scaled-Down c-Si and c-SiGe Wagon-Wheels for the Visualization of the Anisotropy and Selectivity of Wet-Chemical Etchants
Source: Nanoscale Res Lett. 2019 Aug 19;14:285. doi: 10.1186/s11671-019-3114-8 (PMC6701794; doi:10.1186/s11671-019-3114-8)

**Additional file**

S1 Anisotropic etch rate ratio (R_(110)_/R_(100)_) as a function of temperature.

Anisotropic etch rate ratios, R_(110)_/R_(100)_, for various temperatures and TMAH concentrations (in wt.%) extracted from other studies [Tabata, 1992; Sato, 1999; Shikida, 2000 & 2001; Pal, 2009]. In all reported cases of etching of c-Si in TMAH, there seems to be a negative correlation between the anisotropic etch rate ratio, R_(110)_/R_(100)_ and the temperature.


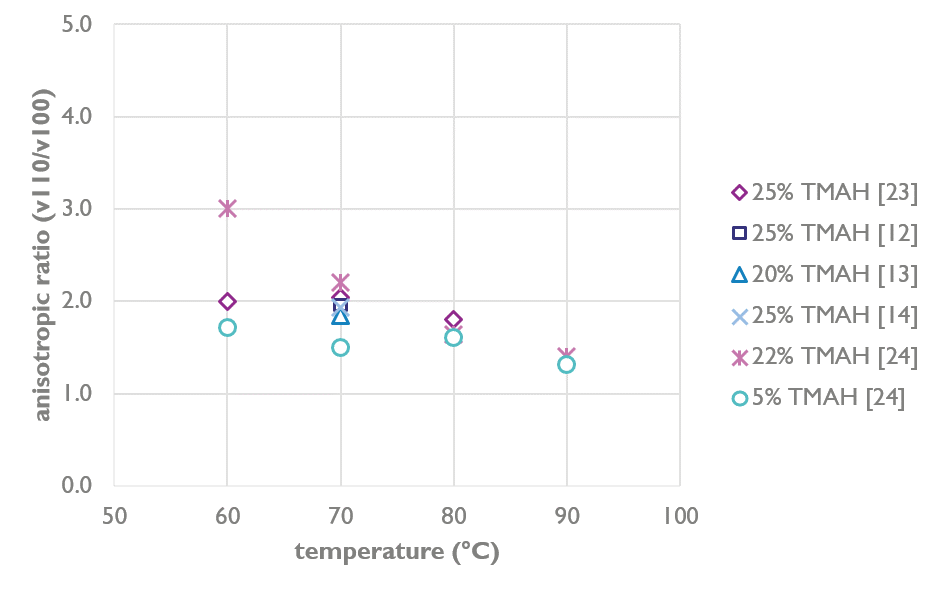


S2 Spoke retraction and onset of the amplification effect.

This schematic illustrates the gradual wet etching of a wagon-wheel spoke as a function of the etching time and the onset of the spoke retraction. The amplification effect initiates only after the spoke tip is sharpened (b), after which the retraction length (∆l) can be used as an approximation for the sidewall loss (∆w), and thus for the etch rate of the sidewall plane. Before situation (b), the etch rate can only be calculated by direct measurement of the sidewall loss (∆d).

**
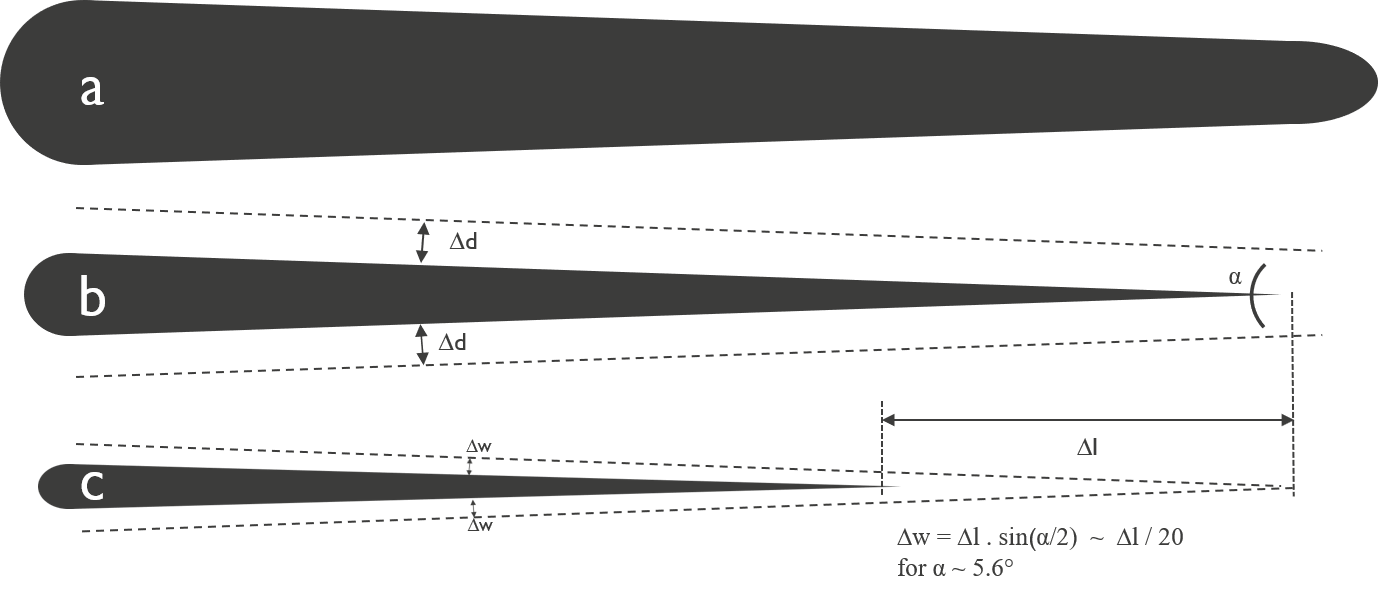
**

(a) Top view of the spoke before wet etching. (b) Spoke after wet etching resulting in sidewall loss (∆d) and in tip sharpening. The dotted line in ‘b’ represents the starting spoke contour of spoke ‘a’. Note that the spoke length did not significantly decrease. (c) Further wet etching results in a significant spoke length retraction (∆l) that can be correlated to the sidewall loss (∆w) by the formula ∆w = ∆l . sin(α/2). The dotted line in ‘c’ represents the contour of spoke ‘b’.

S3.1 Etching time series of c-Si_75_Ge_25_(110) wagon-wheels in PAA-solution.


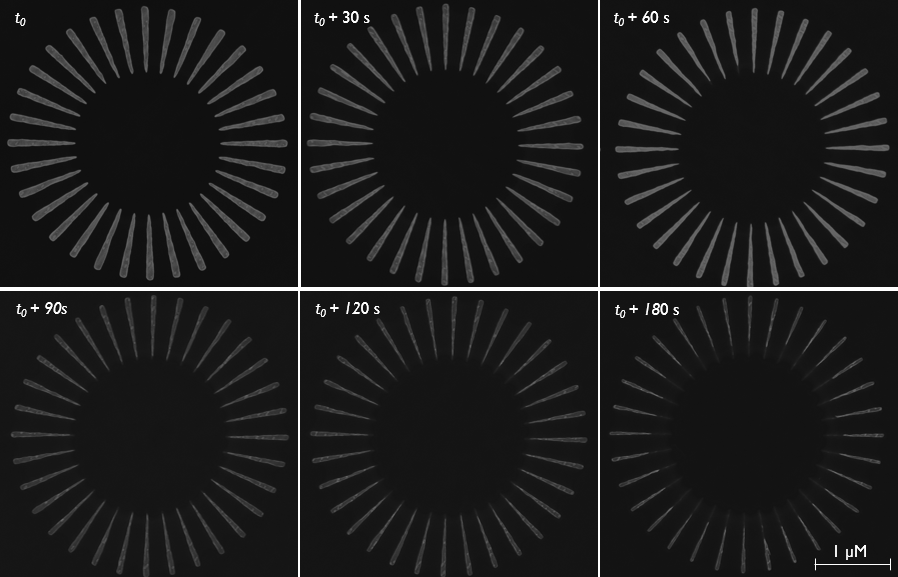


S3.2 Etching time series of c-Si(100) wagon-wheels in PAA-solution.


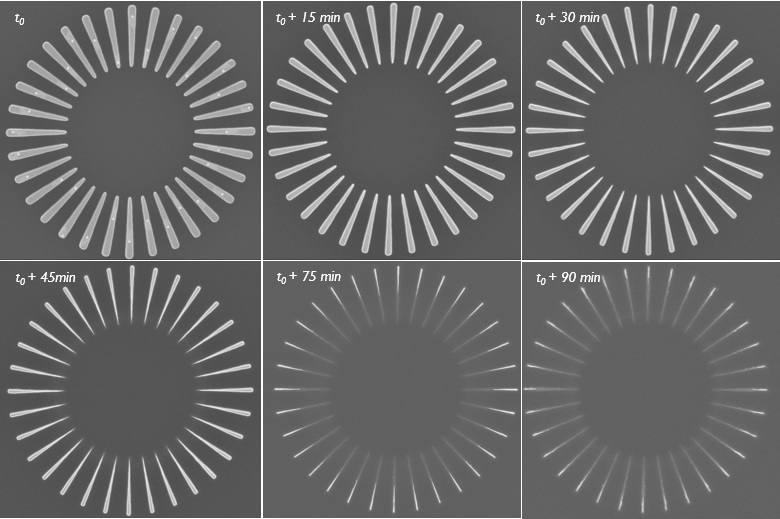

Supplement: Supplementary file 1 — S1. Anisotropic etch rate ratio (R(110)/R(100)) as a function of temperature. Anisotropic etch rate ratios, R(110)/R(100), for various temperatures and TMAH concentrations (in wt.%) extracted from other studies [Tabata, 1992; Sato, 1999; Shikida, 2000 & 2001; Pal, 2009]. In all reported cases of etching of c-Si in TMAH, there seems to be a negative correlation between the anisotropic etch rate ratio, R(110)/R(100) and the temperature. S2. Spoke retraction and onset of the amplification effect. This schematic illustrates the gradual wet etching of a wagon-wheel spoke as a function of the etching time and the onset of the spoke retraction. The amplification effect initiates only after the spoke tip is sharpened (b), after which the retraction length (∆l) can be used as an approximation for the sidewall loss (∆w), and thus for the etch rate of the sidewall plane. Before situation (b), the etch rate can only be calculated by direct measurement of the sidewall loss (∆d). (a) Top view of the spoke before wet etching. (b) Spoke after wet etching resulting in sidewall loss (∆d) and in tip sharpening. The dotted line in ‘b’ represents the starting spoke contour of spoke ‘a’. Note that the spoke length did not significantly decrease. (c) Further wet etching results in a significant spoke length retraction (∆l) that can be correlated to the sidewall loss (∆w) by the formula ∆w = ∆l . sin(α/2). The dotted line in ‘c’ represents the contour of spoke ‘b’. S3.1. Etching time series of c-Si75Ge25(110) wagon-wheels in PAA-solution. S3.2. Etching time series of c-Si(100) wagon-wheels in PAA-solution. (DOCX 495 kb) [file 11671_2019_3114_MOESM1_ESM.docx]
